# Working toward Healthy and Sustainable Diets: The “Double Pyramid Model” Developed by the Barilla Center for Food and Nutrition to Raise Awareness about the Environmental and Nutritional Impact of Foods

**DOI:** 10.3389/fnut.2015.00009

**Published:** 2015-05-04

**Authors:** Luca Fernando Ruini, Roberto Ciati, Carlo Alberto Pratesi, Massimo Marino, Ludovica Principato, Eleonora Vannuzzi

**Affiliations:** ^1^Barilla G.e R. Fratelli S.p.A., Parma, Italy; ^2^Roma Tre University, Rome, Italy; ^3^Life Cycle Engineering, Torino, Italy; ^4^Sapienza – Università di Roma, Rome, Italy; ^5^Roma Tre University, Rome, Italy

**Keywords:** sustainability, diet, Mediterranean, carbon footprint, ecological footprint

## Abstract

The Barilla Center for Food and Nutrition has produced an updated version of the traditional food pyramid based on the Mediterranean diet in order to assess the simultaneous impact that food has on human health and the environment. The Double Pyramid Model demonstrates how the foods recommended to be consumed most frequently are also those exerting less environmental impact, whereas the foods that should be consumed less frequently are those characterized by a higher environmental impact. The environmental impacts resulting from three different menus were compared. All menus were equally balanced and comparable in terms of nutrition, but they differed in relation to the presence of absence of animal flesh and animal products. The first dietary pattern (omnivorous) included both animal flesh and products; the second (lacto-ovo-vegetarian) included animal products (eggs and dairy) but no flesh; and the third (vegan) was solely plant-based. The results obtained suggest that a diet based on the principles of the Mediterranean diet, as suggested by the Double Pyramid, generates a lower environmental impact compared to diets that are heavily based on daily meat consumption.

## Introduction: Why do We Need Sustainable Diets?

It is well-known that our food choices have a significant impact on our health and on the environment. Agriculture is responsible for more than 30% of the global greenhouse gas (GHG) emission when both direct and indirect emissions from land use are considered (1). The livestock sector alone accounts for 18% of the anthropogenic GHG emissions and 80% of total land use (2, 3), as well as being one of the main drivers of deforestation, biodiversity loss, and land degradation (3, 4). In Europe, food consumption accounts for 20–30% of the total ecological impact of households (5).

Despite the extent of world food production, 805 million people were estimated to be chronically undernourished in the period spanning 2012–2014 (6), while 2.1 billion people were considered overweight or obese (7). As a result of the interaction of various factors, including urbanization and increasing prosperity, many countries are experiencing a “nutrition transition” that has led the populations to consume a diet characterized by higher intakes of animal proteins, processed foods, hydrogenated fats, and a lower intake of fiber (8, 9). These dietary changes are causing obesity rates to escalate and increasing the risk of chronic non-communicable diseases (NCD), which currently cause more deaths than all other causes of death combined (10). NCD deaths are projected to reach up to 52 million by 2030 (10) and to account for two-thirds of the global burden of disease if the current dietary trends continue (11, 12). The need to find cost-effective solutions for addressing these environmental and nutritional issues has led to a growing interest in identifying strategies aimed at influencing food consumption, with the scope of promoting healthy and environmentally friendly diets. In 2010, the Food and Agriculture Organization (FAO) together with Biodiversity International emphasized the importance of “sustainable diets,” thus acknowledging the close link between human health and that of our ecosystems (13). The FAO defined sustainable diets as:

[⋯ ]diets with low environmental impacts which contribute to food and nutrition security and to healthy life for present and future generations. Sustainable diets are protective and respectful of biodiversity and ecosystems, culturally acceptable, accessible, economically fair and affordable; nutritionally adequate, safe and healthy; while optimizing natural and human resources. [⋯ ] Sustainable diets can address the consumption of foods with lower water and carbon footprints, promote the use of food biodiversity, including traditional and local foods, with their many nutritionally rich species and varieties.

A specific branch of research has been developed over recent years, which focuses on the relationship between food choices, nutrition, and the environment. Generally, studies have found that the dietary patterns with the lowest environmental impacts are those centered on the consumption of a diverse range of plant foods, while the intake of meat, fish, and animal products is generally correlated with high emissions of greenhouse gases (2, 14–19). Despite the avid interest in sustainable diets within the academic world, greater public awareness is still required. Governments, health councils, and nutritional institutes have started to add sustainability concerns to the traditional food-based dietary guidelines, and to advise the general population on diets that are both good for health and good for the environment. In France, Germany, Sweden, Belgium, and UK, national agencies and NGOs have created the so-called “Sustainable Dietary Guidelines” in an attempt to reconcile nutritional advice with environmental concerns (20–25). Moreover, the Nordic Council of Ministers has provided an estimate of the nutritional changes required in order to achieve more sustainable dietary patterns (26), and the Health Council of the Netherlands has provided its government with recommendation based on available evidence regarding the health and environmental impacts of different foods (27). In Italy, the Barilla Center for Food and Nutrition (BCFN) has developed the “Double Pyramid Model,” a pictorial representation of the extent to which different food groups contribute toward a healthy diet and their environmental impact (28). The purpose of the present study is to present the BCFN’s “Double Pyramid Model” in order to raise people’s awareness of the environmental impact of food consumption.

## The Double Pyramid Model

### General description

The “Double Food and Environmental Pyramid” developed by the Barilla Center for Food and Nutrition is a visual representation that arranges foods according to their contribution to a healthy diet and their environmental impact (28). The Food Pyramid on the left is based on the principles of the Mediterranean diet, which has been explicitly cited by the FAO as an exemplary Sustainable Diet (13) and whose nutritional value has been recognized since the middle of the twentieth century (29, 30). The Mediterranean diet is rich in vegetables, fruits, nuts, unrefined grain cereals, with some fish and limited amounts of red meat and saturated fats (31). Many studies have consistently confirmed that high adherence to the Mediterranean diet can lead to tangible health benefits, including a reduction in the overall mortality rate (30) and a reduced incidence of cardiovascular diseases (31–34), metabolic conditions (35), and certain oncological pathologies (36). The Mediterranean diet has frequently been represented in pyramid form (37–48). The largest part of the pyramid, the base, shows how a well-balanced diet should be primarily based on the consumption of plant foods, while the apex of the pyramid, its smallest part, indicates the foods, which should be consumed more restrictively. After more than 50 years of research, UNESCO has recognized the Mediterranean diet as an intangible cultural heritage of humanity (41).

The environmental pyramid, on the other hand, reclassifies food in terms of the relative magnitude of its environmental impact; thus producing an upside-down pyramid with the most environmentally damaging foods represented at the top, and largely mirroring the order of foods in the adjacent Food Pyramid. The Double Pyramid clearly communicates the inverse relationship between nutritionally recommended foods and their environmental impact.

### Methods and data sources

#### Food and environmental pyramids

The Food Pyramid provides a summary of the various internationally produced guidelines regarding the Mediterranean diet (38, 39, 42). It arranges food according to the relative amount in which they should be consumed, while adhering to the principles of the Mediterranean diet: thus fruit, vegetables, and cereals are found in the bottom half of the pyramid, while red meat, sugars, and fats are at the top (28). The key message conveyed by the Food Pyramid is that our diet should be based mainly on foods of plant origin, as they are rich in vitamins, minerals, fiber, complex carbohydrates, water, and plant proteins, while consumption of the foods residing toward the top of the pyramid should be minimal, being high in saturated fats and simple sugars. The recommended daily intake for each food type was obtained from the “Guidelines for a Healthy Italian Diet” (42), a document published by the Italian Center for Research on Foods and Nutrition (CRANUT).

Life cycle assessment (LCA) methodology was used to generate an estimate of the environmental impact of each food type considered. LCA is an objective technique for assessing the energy consumption and environmental load of a process (which could be an activity or a service), taking into account the whole production chain (28). The results were communicated through three different environmental indicators (28):

- Carbon footprint, which quantifies the greenhouse gas emissions responsible for climate change in terms of amount of CO_2_ equivalents;
- Water footprint (or virtual water content) – calculated as the total volume of freshwater consumed to produce the specific type of food;
- Ecological footprint – a composite indicator (employing conversion factors and specific equivalencies) that measures the anthropogenic impact by considering the different ways in which environmental resources are used. It is measured in terms of global hectares or square meters and is calculated as the sum of all the cropland, grazing land, forest, and fishing grounds required to (i) produce the food and energy required for human activities; (ii) absorb all wastes emitted; and (iii) provide sufficient space for infrastructure.

**Figure 1:**
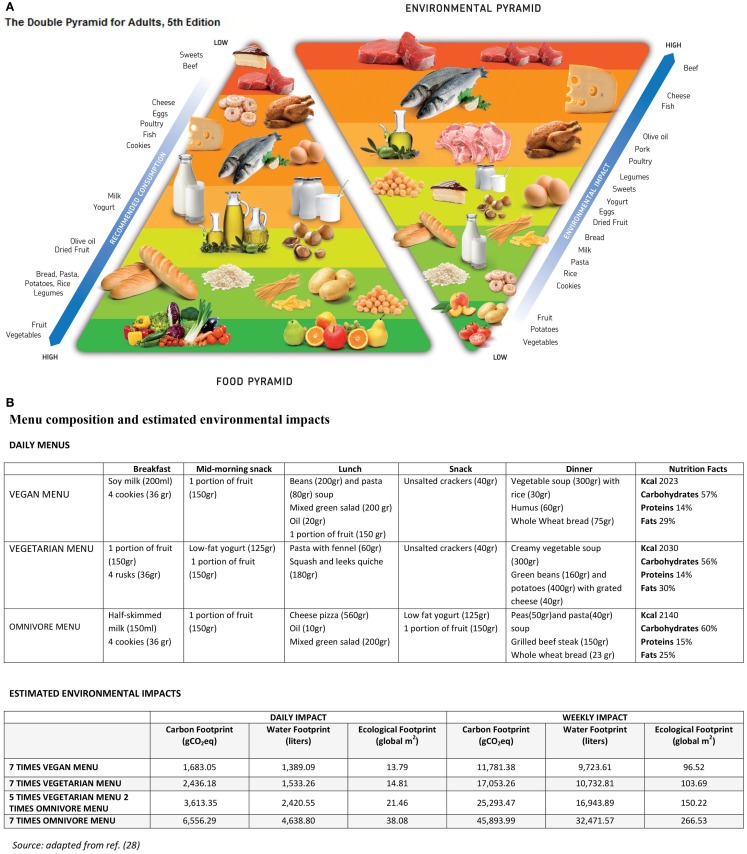
**(A)** The double food and environment pyramid [Source: Ref. (28)]. **(B)** The environmental impact of different menus [Source: Ref. (28)].

Data were obtained from publically available databanks (43–45) and scientific research studies (46) and collated into a specific database. For the fifth edition of the BCFN Double Pyramid, 1,180 data were assembled using more than 250 sources. The values obtained for each of the three environmental indicators refer to 1 kg (or liter) of food. The results for each of the environmental indicators considered are presented in the form separate environmental pyramids (28). However, in order to provide a more effective communications tool, only the Ecological Footprint was used as the reference index when creating the Environmental Pyramid (Figure 1A). The Ecological Footprint was chosen because the unit of measure is easier to visualize and understand compared to those of the other indicators. Moreover, it considers several environmental impact factors simultaneously (46).

#### BCFN daily menus

The Double Pyramid provides consumers with a tool to them decide what to eat on a daily basis, taking into account both health and sustainability concerns. In order to give a practical example of the concept expressed by the Double Pyramid, BCFN has assessed the environmental impact of a series of different dietary regimes. The aim of the comparison, the results of which were published in the latest BCFN publication on the Double Pyramid, was to communicate that even moderate changes in dietary habits can lead to significant benefits in terms of environmental impact.

The comparison included an omnivorous menu, a lacto-ovo-vegetarian menu, and a vegan menu. Each menu was well balanced from a nutritional viewpoint, providing a daily intake of 2,000 calories and similar macronutrient profiles. Each menu coincided with the daily servings of fruits and vegetables as recommended by the Italian CRANUT, including at least three serving of vegetables and two of fruit (42). The omnivorous menu included animal flesh, animal products, and food of plant origin. The lacto-ovo-vegetarian dietary pattern included plant food and animal products, while excluding animal flesh. Finally, the vegan menu reflected a solely plant-based diet, excluding all foods of animal origin (Figure 1B).

## Results and Discussion

### The double pyramid model

The combined pyramids do not provide specific recommendations for food choices that are both healthy and sustainable, but they provide a unified model of the connection between the twin goals of health and environmental protection. Indeed, the Double Pyramid shows that the foods offering the greatest benefits from a nutritional viewpoint (such as vegetables, grains, pulses, and fruit) are those with the lowest environmental impact, while the foods that should be consumed in moderation for health reasons, such as red and processed meats, are those with the highest impact (Figure 1A). While some of the data used to compile the pyramids are still the subject of debate within the scientific community and while the sustainability of fishing remains a concern (28), the pyramids generally coincide with the majority of health and ecological data present in the scientific literature. The Environmental Pyramid demonstrates that vegetables (and plant foods overall) have an environmental impact that is lower than products of animal origin. Similarly, the water footprint of 1kg of bovine meat (18,870.l) is 61 times higher than the water footprint of the same amount of vegetables (310.l), and 11 times higher than the water footprint of pasta (1,770.l). Similarly, the carbon footprint of fruit (475 g CO_2_eq) and vegetables (820 g CO_2_eq) are 55 and 32 times lower, respectively, than the carbon footprint of red meat (26,170 g CO_2_eq) (28).

It is important to specify that although the Double Pyramid refers to the Mediterranean diet, this does not necessarily mean that it is the only well-balanced dietary regime. Over the decades, many governments have developed a variety of graphic tools with the scope of informing and educating people about how to follow a well-balanced diet in order to be healthy. Such guidelines have been developed according to the typical dietary regimes of the population, focusing on traditionally consumed and locally accessible foods. However, despite minor differences due to specific cultural aspects or the availability of certain food types, all of these diets agree on the fact that a well-balanced dietary regime should be mainly composed of fruit, vegetables, cereals (especially whole grain), and legumes, while the consumption of red meat, fats, and sugars should be limited (20, 38, 47, 48). Due to the versatility of the Double Pyramid Model, it can be easily adapted to different cultures and modified accordingly.

### Environmental impact of the different menus

With regard to the environmental impact of the different menus examined, both the vegetarian and the vegan dietary patterns performed better than the omnivorous one. On average, the vegetarian menu has an environmental impact that is 2.8 times lower than the omnivore menu, while the impact of the vegan menu is 3.3 times lower. The carbon footprint of the vegan menu is equal to 1,683.05 g CO_2_eq per person per day, compared to the 2436.18 g CO_2_eq for the vegetarian menu and 6.556,2 g CO_2_eq for the omnivorous menu. The water footprint of the vegetarian menu was 1,533.26 l of water per person per day, which was almost 2.5 times less than the omnivorous menu (4,638.80), but higher than the vegan menu (1,389.09). As regards the ecological footprint, the omnivore menu has an environmental impact that is 2.57 times higher than the vegetarian one: 38.08 (global) m^2^ vs. 14.81 m^2^ per person per day, respectively, a difference of up to 23 m^2^ per day or 162 m^2^ per week – a large quota in the daily impact of an individual. The difference is even higher when considering the vegan menu (2.8 times; 24.3 m^2^ per day higher) (Figure 1B).

These data allow us to estimate the potential reduction that could be achieved in an individual’s environmental footprint by changing eating habits. By analyzing the average amount of food consumed by a male adult in a week, we identified four different menus according to how often the menu is based on the consumption of animal protein (flesh and animal products). As illustrated in Figure 1B, the vegan diet has by far the lowest environmental impact. The results of this analysis are in line with the message conveyed by the Double Pyramid, as the dietary patterns richer in vegetables and plant foods are those with the lowest environmental impact. While the acceptance of a solely plant-based diet may be difficult for some (18, 48), our infographic tool clearly denotes the benefits of incorporating a semi-vegetarian, vegetarian, or vegan diet into our eating routines and offers an added incentive for those trying to improve their health in terms of a reduced strain upon environmental resources. Our analysis demonstrates that it would be possible to achieve modest environmental results without having to completely cut animal flesh and products out of the diet. By limiting meat consumption to just twice a week – an amount that is in line with the recommendations established by CRA-NUT (42) – it would be possible to “save” up to 16.6 global square meters, 2,218 l of water, and 2,942 g of carbon dioxide per person per day (Figure 1B) (28).

## Conclusion

Current food consumption patterns in industrialized countries are having a detrimental impact on both human health and the environment. In this context, it is essential to raise public awareness concerning the environmental and nutritional impacts of our food choices. The most interesting result emerging from the Double Pyramid Model is the strong correlation between the environmental impact of food and their nutritional characteristics. Specifically, it has been demonstrated that the foods whose consumption should be moderated for health reasons are also those that have a greater impact in terms of soil use, water consumption, and CO_2_ emission. In other words, to achieve a sustainable, healthy diet is essential to eat more plant-based foods and reduce our consumption of meat, animal products, and other foods, like salted snacks and sweets, which offer little in terms of nutritional value.

Here, in order to estimate the extent to which an individual’s food choices can influence their environmental impact, three dietary regimes were analyzed. All the menus were balanced from the nutritional perspective, but they differed in relation to the amount of animal products included. The solely plant-based diet shows the best results in terms of environmental impact, out-performing both the vegetarian and the omnivorous diets. Even adopting a semi-vegetarian diet (that is, maintaining an omnivore diet only twice a week) offers individuals with the possibility of reducing their environmental impact compared to that generated from a dietary regime rich in animal products. By limiting the intake of animal flesh to just twice a week, it would be possible for an individual to reduce his environmental impact, generated by food consumption, by up to one-third.

## Conflict of Interest Statement

The authors declare that the research was conducted in the absence of any commercial or financial relationships that could be construed as a potential conflict of interest.

## References

[1] Bellarby J Foereid B Hastings A Smith P Cool Farming: Climate Impacts of Agriculture and Mitigation Potential. Amsterdam: Greenpeace (2008).

[2] Stehfest E Bouwman L van Vuuren D den Elzen M Eickhout B Kabat P Climate benefits of changing diet. Clim Change (2009) 95:83–102 10.1007/s10584-008-9534-6

[3] Steinfeld H Gerber P Wassenaar T Castel V Rosales M de Haan C Livestock Long Shadow: Environmental Issues and Options. Rome: FAO (2006).

[4] Garnett T What is a Sustainable Healthy Diet? A Discussion Paper. Oxford: Food Climate Research Network (2014).

[5] Tukker A Goldbohm A de Koning A Verheijden M Kleijn R Wolf O Environmental impacts of changes to healthier diets in Europe. Ecol Econ (2011) 70:1776–88 10.1016/j.ecolecon.2011.05.001

[6] FAO, IFAD, WFP. The State of Food Insecurity in the World 2014. Strengthening the Enabling Environment for Food Security and Nutrition. Rome: FAO (2014).

[7] Ng M Fleming T Robinson M Thomson B Graetz N Margono C. Global, regional, and national prevalence of overweight and obesity in children and adults during 1980-2013: a systematic analysis for the Global Burden of Disease Study 2013. Lancet (2014) 9945:766–81. 10.1016/S0140-6736(14)60460-8 24880830 PMC4624264

[8] Popkin BM. Global nutrition dynamics: the world is shifting rapidly toward a diet linked with noncommunicable diseases. Am J Clin Nutr (2006) 84:289–98. 16895874 10.1093/ajcn/84.1.289

[9] Popkin BM Adair LS Ng SW. Global nutrition transition and the pandemic of obesity in developing countries. Nutr Rev (2012) 70:3–21. 10.1111/j.1753-4887.2011.00456.x 22221213 PMC3257829

[10] World Health Organization. Global Status Report on Non-Communicable Diseases 2014. Geneva: World Health Organization (2014).

[11] Chopra M Galbraith S Darnton-Hill I. A global response to a global problem: the epidemic of overnutrition. Bull World Health Organ (2002) 80:952–8. 12571723 PMC2567699

[12] Nishida C Uauy R Kumanyika S Shetty P. Diet, nutrition and the prevention of chronic diseases: report of a joint WHO/FAO expert consultation. Public Health Nutr (2004) 7:245–50. 10.1079/PHN2003592 14972063

[13] FAO. Sustainable Diets and Biodiversity. Rome: FAO (2010).

[14] Macdiarmid J Kyle J Horgan G Loe J Fyfe C Johnstone A Livewell: A Balance of Healthy and Sustainable Food Choices. WWF Report. Aberdeen: Rowett Institute of Nutrition and Health, University of Aberdeen (2011).

[15] Tilman D Clark M. Global diets link environmental sustainability and human health. Nature (2014) 515(7528):518–22. 10.1038/nature13959 25383533

[16] Sáez-Almendros S Obrador B Bach-Faig A Serra-Majem L. Environmental footprints of Mediterranean versus Western dietary patterns: beyond the health benefits of the Mediterranean diet. Environ Health (2013) 12:118. 10.1186/1476-069X-12-118 24378069 PMC3895675

[17] Westhoek H Lesschen JP Rood T Wagner S De Marco A Murphy-Bokern D Food choices, health and environment: effects of cutting Europe’s meat and dairy intake. Glob Environ Change (2014) 26:196–205 10.1016/j.gloenvcha.2014.02.004

[18] Van Dooren C Marinussen M Blonk H Aiking H Vellinga P Exploring dietary guidelines based on ecological and nutritional values: a comparison of six dietary patterns. Food Policy (2014) 44:36–46 10.1016/j.foodpol.2013.11.002

[19] Baroni L Cenci L Tettamanti M Berati M. Evaluating the environmental impact of various dietary patterns combined with different food production systems. Eur J Clin Nutr (2006) 61(2):279–86. 17035955 10.1038/sj.ejcn.1602522

[20] Westland S Crawley H Healthy and Sustainable Diets in the Early Years. London: First step nutrition Trust (2012).

[21] Agence de l’Environnement et de la Maîtrise de l’Energie (ADEME). Mes Achats. ADEME (2012). Available form: http://www.ademe.fr/particuliers-eco-citoyens/achats/alimentation

[22] German Council of Sustainable Development. The Sustainable Shopping Basket. Berlin: German Council of Sustainable Development General Secretariat (2013).

[23] Reddy S Lang T Dibb S Setting the Table. London: Sustainable Development Commission (2009).

[24] National Food Administration and Environmental Protection Agency. Towards Environmentally Sound Dietary Guidelines – Scientific Basis for Environmental Assessment of the Swedish National Food Agency’s Dietary Guidelines. NFA (2013). Available from: http://www.slv.se/upload/dokument/rapporter/mat_miljo/2008_NFA_report_9_towards_environmentally_sound_dietary_guidelines.pdf

[25] Bruxelles Environment. Nutrition and the Environment. Bruxelles: Bruxelles Environnement, IBGE (2013).

[26] Norden. Nordic Nutrition Recommendations 2012. Copenhagen: Nordic Council of Ministers (2014).

[27] Health Council of the Netherlands. Guidelines for a Healthy Diet: The Ecological Perspective. The Hague: Health Council of the Netherlands (2011).

[28] Barilla Center for Food & Nutrition. Double Pyramid 2014. Fifth Edition: Diet and Environmental Impact. Parma: BCFN (2014).

[29] Keys A Aravanis C Blackburn HW Van Buchem FSP Buzina R Djordjevic BS Epidemiologic studies related to coronary heart disease: characteristics of men aged 40-59 in seven countries. Acta Med Scand (1967) 460:1–392. 5226858

[30] Keys A Seven Countries. A Multivariate Analysis of Death and Coronary Heart Disease. Cambridge: Harvard University Press (1980).

[31] Trichopoulou A Costacou T Bamia C Trichopoulos D Adherence to a Mediterranean diet and survival in a Greek population. N Engl J Med (2003) 348:2599–608 10.1056/NEJMoa025039 12826634

[32] Fung TT Rexrode KM Mantzoros CS Manson JE Willett WC Hu FB. Mediterranean diet and incidence of and mortality from coronary heart disease and stroke in women. Circulation (2009) 119:1093–100. 10.1161/CIRCULATIONAHA.108.816736 19221219 PMC2724471

[33] Lopez-Garcia E Rodriguez-Artalejo F Li TY Fung TT Li S Willett WC The Mediterranean-style dietary pattern and mortality among men and women with cardiovascular disease. Am J Clin Nutr (2014) 99:172–80. 10.3945/ajcn.113.068106 24172306 PMC3862454

[34] Estruch R Ros E Salas-Salvado J Covas MI Corella D Aros F Primary prevention of cardiovascular disease with a Mediterranean diet. N Engl J Med (2013) 368:1279–90. 10.1056/NEJMoa1200303 29897867

[35] Babio N Toledo E Estruch R Ros E Martínez-González M Castañer O. Mediterranean diets and metabolic syndrome status in the PREDIMED randomized trial. Can Med Assoc J (2014) 186(17):E649–57. 10.1503/cmaj.140764 25316904 PMC4234734

[36] Couto E Boffetta P Lagiou P Ferrari P Buckland G Overvad K. Mediterranean dietary pattern and cancer risk in the EPIC cohort. Br J Cancer (2011) 104(9):1493–9. 10.1038/bjc.2011.106 21468044 PMC3101925

[37] Willett WC Sacks F Trichopoulou A Drescher G Ferro-Luzzi A Helsing E Mediterranean diet pyramid: a cultural model for healthy eating. Am J Clin Nutr (1995) 61(6):1402S–6S. 7754995 10.1093/ajcn/61.6.1402S

[38] World Health Organization Europe. Food Based Dietary Guidelines in the WHO European Region. Copenhagen: World Health Organization (WHO) (2003).

[39] Oldways Preservation & Exchange Trust. Mediterranean Diet Pyramid. Oldways Preservation & Exchange Trust (2009). Available form: http://oldwayspt.org/resources/heritage-pyramids/mediterranean-pyramid/overview

[40] Barilla Center for Food & Nutrition. Double Pyramid: Healthy Food for People, Sustainable Food for the Planet. Milan: Barilla Center for Food & Nutrition (2010).

[41] Saulle R La Torre G The Mediterranean diet, recognized by UNESCO as a cultural heritage of humanity. Ital J Public Health (2010) 7(4):414–5.

[42] Center for Research on Foods and Nutrition (CRA-NUT). Dietary Guidelines for a Healthy Italian Diet. Rome: CRANUT (2003).

[43] Ecoinvent Database. Available from: http://www.ecoinvent.org

[44] Environmental Product Declaration Database. Available from: www.environdec.com

[45] LCA Food Database. Available from: www.LCAfood.dk

[46] Ewing B Moore D Goldfinger S Oursler A Reed A Wackernagel M The Ecological Footprint Atlas 2010. Oakland, CA: Global Footprint Network (2010).

[47] Dwyer JT Bury E Dietary guidelines around the world: regional similarities and differences and new innovations. In: Berdanier C Dwyer J Heber D, editors. Handbook of Nutrition and Foods – Third Edition. Boca Raton, FL: CRC Press (2014). p. 447–68.

[48] Dibb S Fitzpatrick I Let’s Talk About Meat: Changing Dietary Behaviour for the 21st Century. London: Eating Better (2014).

